# Co-cultivation rescues suicidal *Paenibacillus amylolyticus* swarms

**DOI:** 10.1093/ismejo/wraf225

**Published:** 2025-10-09

**Authors:** Dana Ronin, Mads Frederik Hansen, Mette Burmølle

**Affiliations:** Section of Microbiology, Department of Biology, University of Copenhagen, Universitetsparken 15, 2100, Copenhagen Ø, Denmark; Section of Microbiology, Department of Biology, University of Copenhagen, Universitetsparken 15, 2100, Copenhagen Ø, Denmark; Section of Microbiology, Department of Biology, University of Copenhagen, Universitetsparken 15, 2100, Copenhagen Ø, Denmark

**Keywords:** biofilm, swarming, interspecies interactions, ecological suicide, pH stabilization

## Abstract

Bacterial locomotion is integral to acquiring resources and getting access to new niches. Swarming, a type of motility where flagellated bacteria cooperatively move together across a semi-solid surface, is one example of how bacteria can colonize new territories. This collective behavior is temporally and spatially orchestrated, requiring task specialization of community members. In this study, we paired a swarming bacterium, *Paenibacillus amylolyticus*, with a non-swarmer, *Stenotrophomonas maltophilia*, to investigate the impact on fitness of each strain. In dual-species conditions, the community swarm became significantly thicker and improved the ability of *S. maltophilia* to range into new territories. Swarming enabled *P. amylolyticus* to cross barriers of antimicrobials, whereas the thicker, dual-species swarm did not empower *S. maltophilia* to cross. Comparative studies of population dynamics revealed that over time, monospecies swarms of *P. amylolyticus* entered a state unable to grow despite still showing reductase activity. However, in a dual-species swarm, *S. maltophilia* rescued *P. amylolyticus* from this state. This rescue is attributed to the pH stabilization that occurs in this two-species combination, where *S. maltophilia* alkalizes the environment, thereby providing a more favorable environment for *P. amylolyticus*.

For many living organisms, locomotion is an essential aspect of life. The ability to migrate according to environmental conditions and stimuli enables positioning in favorable habitats and determines success [1, 2]. In nature, cells are often organized in multicellular populations that require coordinated behavior. One such example is swarming, in which flagellated bacteria produce biosurfactants to reduce surface tension and collectively migrate through a thin film [2]. This collective behavior facilitates expansion over nutrient sources and translocation to new ecological niches. Over time, swarm expansion is a dynamic process with a high level of heterogeneity, cell–cell contact, and intercellular interactions [3, 4]. Most studies have focused on single species swarm development; however, bacteria typically live in multispecies environments [5]. Given previously described interspecies interactions such as cross-feeding, pH stabilization, and spatial intermixing [6–8], we investigated the effect of pairing swarming *Paenibacillus amylolyticus* with non-swarming *Stenotrophomonas maltophilia.* To evaluate how co-swarming affected the fitness of both members, we measured two key indicators: (i) access to new niches, assessed by surface expansion and antibiotic tolerance and (ii) cell numbers of each member in mono- and dual-species swarms.

Many *Paenibacillus* spp. can swarm [9–11]; one well-known swarming requirement is surfactant production. To verify surfactant production in *P. amylolyticus*, we identified genes with homology to those in the *Bacillus subtilis* surfactin synthetase cluster (Suppl. Fig. 1) [12]. Surfactin is synthesized by non-ribosomal peptide synthesis (NRPS) systems, three of which were previously identified in the *P. amylolyticus* genome [13]; low homology hits for surfactin synthesis may also include related genes from other NRPS systems. Also, the presence of a surface tension-reducing compound in the culture was verified (Fig. 1A). Additionally, swarming motility is powered by rotating flagella movement [2]; we identified lophotrichous flagella in *P. amylolyticus* (Fig. 1B) and observed swarming behavior at various agar concentrations, with reduced swarm speed at higher concentrations (Fig. 1C and D). Once swarming initiated, *P. amylolyticus* formed a thin swarm layer (Suppl. Fig. 2) that spread over the entire agar plate after about 11 h of incubation (Fig. 1D, 0.9% agar). When mixed with *S. maltophilia*, the swarm layer became significantly thicker (Fig. 1E; Suppl. Fig. 2). This thicker part was visibly asymmetric (Fig. 1E), indicating that *S. maltophilia* could perform passive sliding motility, using surfactant as a public good to access new areas [14]. To determine whether *S. maltophilia* was translocated along with *P. amylolyticus*, we sampled throughout the dual-species swarm from the center to the edge. At the swarm periphery, *P. amylolyticus* constituted the forefront of the community, whereas *S. maltophilia* was present solely in the thicker areas of the swarm (Suppl. Fig. 2). Nevertheless, *S. maltophilia* displayed territory expansion when it gained the ability to spread in a dual-species swarm.

**Figure 1:**
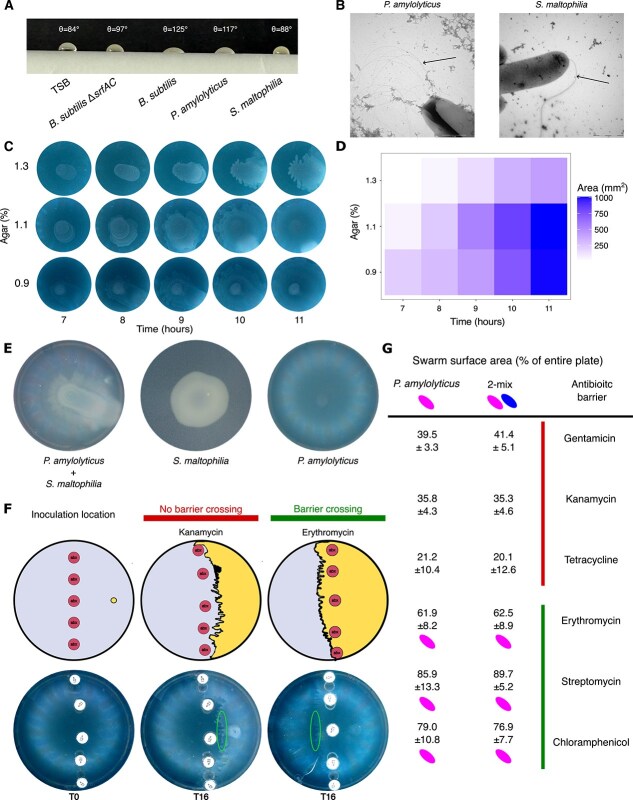
Establishing swarming conditions and the general swarming phenotype. A) The drop collapse assay was used to phenotypically test for surfactant production in *Paenibacillus amylolyticus* and *Stenotrophomonas maltophilia*. A collapsed drop confirms a surfactant present in the culture. Tryptic soy broth (TSB) and *Bacillus subtilis* Δ*srfAC* were included as negative controls, whereas *B. subtilis* wild-type constituted the positive control. The contact angle for each drop (measured externally) was calculated with ImageJ using the theta e values. Contact angles above 110° indicate a collapsed drop. B) The presence of flagella was confirmed in both *P. amylolyticus* and *S. maltophilia* by transmission electron microscopy (TEM). C) Agar percentages ranging from [0.9, 1.1, 1.3] % were tested to establish swarming conditions for *P. amylolyticus*. D) ImageJ was used to calculate the swarm area (mm^2^) over time for each agar percentage tested. E) Visualizing the effect of pairing swarming *P. amylolyticus* with non-swarming *S. maltophilia*. The dual-species swarm expanded over the plate, gaining access to the outer edges of the plate, which was not observed for the monoculture of *S. maltophilia*. There may be sliding motility at play due to the visible asymmetry seen on the plate [14]. The *P. amylolyticus* swarm spreads over the entire plate as a thin monolayer of cells making it hard to visualize. Images were taken after 48 h. F) The abilities of a monospecies swarm (*P. amylolyticus*) and a dual-species swarm (*P. amylolyticus* + *S. maltophilia*) to cross an antibiotic barrier were assessed. Shown here is a schematic illustration of the inoculation point at time 0 h. Examples of a swarm not crossing an antibiotic barrier (e.g., kanamycin) as well as a swarm crossing an antibiotic barrier (e.g., erythromycin) are depicted. The corroborating image is shown below the schematic (taken at 16 h). Locations of swarm restreaks are indicated with a green circle. G) The ability of a swarm to cross six antibiotic disc barriers was assessed: streptomycin (300 μg), erythromycin (15 μg), kanamycin (30 μg), tetracycline (30 μg), gentamicin (10 μg), and chloramphenicol (30 μg). After 16 h, the swarm surface area as a percentage of the entire plate was calculated using ImageJ. Swarm surface area above 50% indicates the swarm crossing the barrier, whereas values less than 50% indicate not crossing. Four biological replicates were done for each antibiotic. Mean values and standard deviations are shown. To test which of the two members were able to survive past the antibiotic barrier, we streaked the biomass which crossed the barrier and tested for the presence of each species using selective plating (streptomycin 5000 μg/mL for *P. amylolyticus* and erythromycin 15 μg/mL for *S. maltophilia*). *P. amylolyticus* presence is indicated as a pink oval and *S. maltophilia* presence as a blue oval. Only *P. amylolyticus* was able to survive past the antibiotic barriers, regardless of whether it was in a mono- or dual-species swarm.

Previous studies have shown that swarm behavior can desensitize the susceptibility to antibiotics [15, 16]. Gaining antibiotic tolerance during motility can provide protection when colonizing new niches. We tested the ability of mono- and dual-species swarms to cross antibiotic barriers. Neither mono- nor dual-species swarms could cross kanamycin, gentamicin, or tetracycline barriers (Fig. 1F,G), which is consistent with the essential swarmer, *P. amylolyticus*, being sensitive to these compounds (Suppl. Fig. 3). For erythromycin, streptomycin, and chloramphenicol barriers, both the mono- and dual-species swarms were able to cross (Fig. 1F,G). *P. amylolyticus* is resistant to streptomycin, but sensitive to erythromycin and chloramphenicol (Suppl. Fig. 3, Suppl. Fig. 4). When determining which members crossed the antibiotic barrier in the dual-species swarms, solely *P. amylolyticus* crossed (Fig. 1G); the presence of *S. maltophilia* in the dual-species swarm did not provide any apparent advantage for antibiotic susceptibility that was not already achieved by the monospecies swarm on its own. *S. maltophilia* could not utilize the *P. amylolyticus*-associated surfactant to expand its reach into new areas, even across barriers for which it has resistance (e.g., kanamycin) (Fig. 1G and F). Although being in a dual-species swarm did not induce further antibiotic tolerance, swarming motility in general benefited *P. amylolyticus*, allowing it to grow in the presence of antibiotics it is normally sensitive to (erythromycin and chloramphenicol).

Having established that swarming resulted in growth area expansion both in presence and absence of antibiotics, we next assessed specific abundances as a second measure of fitness during co-swarming. *P. amylolyticus* and *S. maltophilia* abundances were quantified over time in mono- and dual-species swarms. There was no significant difference between the two conditions for *S. maltophilia* cell counts at any of the respective timepoints (Fig. 2A), indicating no benefit or cost for this species during co-swarming despite the territory expansion previously reported. For 12- and 24-h old swarms, there was no difference in *P. amylolyticus* cell counts between the mono- and dual-species swarms. However, for 72-h old swarms, we observed significantly lower cell counts for the monospecies *P. amylolyticus* swarm compared to the dual-species swarm (Fig. 2A). Thus, instead of competing for shared resources, *P. amylolyticus* required the presence of *S. maltophilia* to uphold a viable population of cells. To test whether *P. amylolyticus* cells were dead after 72 h in the monospecies swarm, we applied a bacterial reductase activity stain, which revealed a plethora of active and vital cells (Fig. 2B), suggesting that they were not dead but had entered a non-growth state.

**Figure 2:**
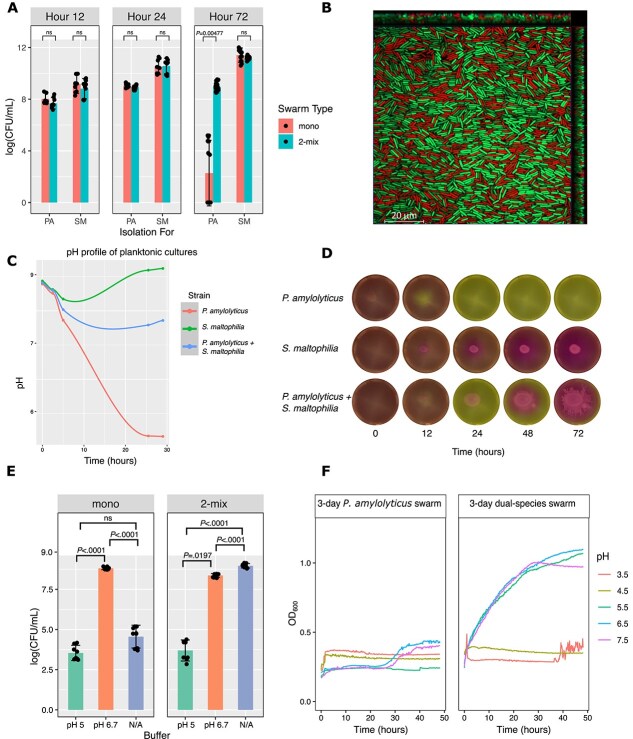
Viability of *P. amylolyticus* linked to pH fluctuations in a dual-species swarm. A) Cell numbers (CFU/mL) of *Paenibacillus amylolyticus* (PA) and *Stenotrophomonas maltophilia* (SM) in mono- and dual-species swarms. CFU/mL counts were done after 12, 24, and 72 h. Individual species quantification was done using selective plating (streptomycin 5000 μg/mL for *P. amylolyticus* and erythromycin 15 μg/mL for *S. maltophilia*). No differences between cell numbers in mono- and dual-species swarms were observed for either species (*P* > 0.05; two-sample t-test), except at 72 h for *P. amylolyticus*. There was a significant increase in *P. amylolyticus* cells when in a dual-species swarm compared to a monospecies swarm (*P* = 0.00477; Wilcoxon rank sum test with continuity correction). B) A confocal scanning laser microscopy (CLSM) image of *P. amylolyticus* swarm stained with the *Bac*Light RedoxSensor vitality stain and propidium iodide. Scale bar indicates 20 μm. The image was taken 25 mm from the swarm center. C) pH quantification over time in planktonic cultures of *P. amylolyticus*, *S. maltophilia*, and *P. amylolyticus* + *S. maltophilia*. D) Phenol red pH indicator was added to swarming plates for *P. amylolyticus*, *S. maltophilia*, and *P. amylolyticus* + *S. maltophilia*. Yellow indicates a pH of below 6.4, whereas pink indicates a pH above 8. Images were recorded over time with the Reshape Imaging System (RIS) robot. E) Cell numbers (CFU/mL) of *P. amylolyticus* in mono- and dual-species swarms on buffered and unbuffered plates (indicated by pH 5.0, pH 6.7, and N/A, respectively). To test if *P. amylolyticus* rescue during swarming was pH mediated, swarming plates were buffered to pH 6.7; these plates allowed *P. amylolyticus* from a monospecies swarm to survive significantly better compared to unbuffered plates (*P* <0.0001), whereas there was no significant difference in cell counts between pH 5.0 buffered plates and unbuffered plates. In the swarm plates buffered to pH 5.0, *P. amylolyticus* from a dual-species swarm survived worse compared to the unbuffered plates (*P* <0.0001). There was also a significant difference between the pH 6.7 buffered plates and unbuffered plates for the dual-species swarm (*P* <0.0001) (mixed model Geisser-Greenhouse correction with Tukey multiple comparison). F) Regrowth of *P. amylolyticus* cells sampled from three-day old swarms (mono- and dual-species) in TSB media with pH ranging from 3.5 to 7.5 over time.

A previous study found that *Xanthomonas perforans* modulated the swarming behavior of *Paenibacillus* spp. by pH fluctuations [11]. Given that *P. amylolyticus* and *S. maltophilia* have pH stabilization attributes when together (Fig. 2C) [8], we applied phenol red to monitor pH changes over time during swarming. When *P. amylolyticus* was swarming independently, it greatly acidified its environment after 24 h (Fig. 2D), meaning that 72-h old swarms were exposed to an acidic environment for an extended period. However, in the dual-species swarm, *S. maltophilia* increased the pH after an initial low pH caused by *P. amylolyticus*; the pH of the swarming plate increased after 24 h, starting from the center and expanding to the edges over time (Fig. 2D). To verify that pH was the key determinant of *P. amylolyticus* survival after 72 h of swarming, swarming plates were buffered to pH 5.0 and pH 6.7. Phenol red was used to confirm the stability of the pH buffer over time, despite bacterial growth (Suppl. Fig. 5). Similarly to co-cultivation with *S. maltophilia*, monospecies swarming plates buffered to pH 6.7 ensured a high abundance of *P. amylolyticus* after 72 h of growth. Conversely, dual-species swarming plates buffered to pH 5.0 reduced the *P. amylolyticus* cell counts to levels comparable to those recorded in unbuffered monospecies swarms (Fig. 2E). Finally, to test if *P. amylolyticus* was rescued after entering its non-growing state, we re-cultured 3-day old *P. amylolyticus* swarms in liquid TSB at different pHs (ranging from 3.5 to 7.5). No significant growth was detected over 48 h, indicating that a change in pH did not rescue the cells. In fact, low pH (pH < 5.5) also prevented regrowth of *P. amylolyticus* cells originating from dual-species swarm communities (Fig. 2F).

“Ecological suicide” has been previously described in *Paenibacillus* sp.; at increasing cell densities, *Paenibacillus* sp. strongly acidifies its environment causing its own extinction [17]. In our study, when *P. amylolyticus* was paired with *S. maltophilia*, the latter raised the pH after a period of low pH (Fig. 2C and D), effectively rescuing *P. amylolyticus* from its self-imposed cytotoxic environment. Our data demonstrate how interspecies interactions may benefit co-culture partners; *P. amylolyticus* directly benefitted from the presence of *S. maltophilia*, which rescued it from extinction. Even though *S. maltophilia* did not benefit in cell numbers when in the dual-species swarm, it co-localized along with *P. amylolyticus* and gained access to new areas. In natural environments, such colony expansion may give access to new niches, supporting growth as well as survival.

## Supplementary Material

figure1_spellingFixedinG_wraf225

supplMaterialCompiled_wraf225

## Data Availability

All data generated or analyzed during this study are included in this published article and its supplementary information files.
