# Supplementary material for: Co-cultivation rescues suicidal Paenibacillus amylolyticus swarms
Source: ISME J. 2025 Oct 9;19(1):wraf225. doi: 10.1093/ismejo/wraf225 (PMC12599307; doi:10.1093/ismejo/wraf225)
Supplement: figure1_spellingFixedinG_wraf225 [file figure1_spellingfixeding_wraf225.pdf]

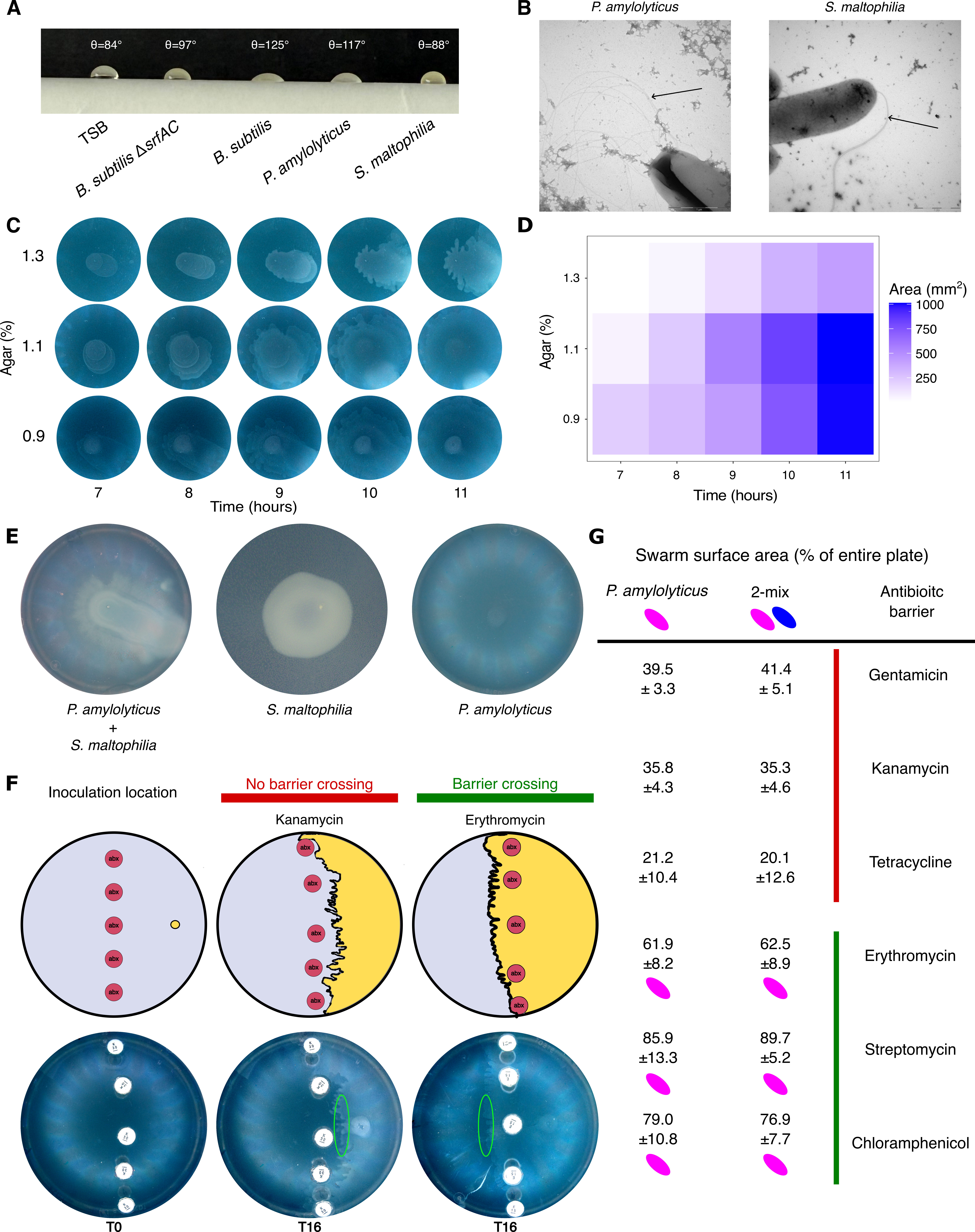
A
θ=84°
θ=97°
θ=125°
θ=117°
θ=88°
TSB
B. subtilis ΔsrfAC
B. subtilis
P. amylolyticus
S. maltophilia
B
P. amylolyticus
S. maltophilia
C
1.3
1.1
0.9
Agar (%)
7
8
9
10
11
Time (hours)
D
1.3
1.1
0.9
Agar (%)
7
8
9
10
11
Time (hours)
Area (mm2)
1000
750
500
250
E
P. amylolyticus + S. maltophilia
S. maltophilia
P. amylolyticus
F
Inoculation location
No barrier crossing
Barrier crossing
Kanamycin
Erythromycin
abx
T0
T16
T16
G
Swarm surface area (% of entire plate)
P. amylolyticus
2-mix
Antibioitc barrier
39.5 ± 3.3
41.4 ± 5.1
Gentamicin
35.8 ±4.3
35.3 ±4.6
Kanamycin
21.2 ±10.4
20.1 ±12.6
Tetracycline
61.9 ±8.2
62.5 ±8.9
Erythromycin
85.9 ±13.3
89.7 ±5.2
Streptomycin
79.0 ±10.8
76.9 ±7.7
Chloramphenicol
