# Supplementary material for: Co-cultivation rescues suicidal Paenibacillus amylolyticus swarms
Source: ISME J. 2025 Oct 9;19(1):wraf225. doi: 10.1093/ismejo/wraf225 (PMC12599307; doi:10.1093/ismejo/wraf225)
Supplement: supplMaterialCompiled_wraf225 [file supplmaterialcompiled_wraf225.pdf]

Material and methods

Bacterial strains and media

All bacteria listed in Table 1 were streaked out onto Tryptic Soy Agar (TSA) plates (3% (W/V) TSB (Millipore), 1.5% (W/V) agar) at 24°C. For planktonic cultures, a colony was picked after 48 h and inoculated into TSB at 250 rpm at 24°C or 30°C.

Table 1. Bacterial strains used in this study.

**Designation**	**Bacteria**	**Notes**	**Source**
Sp949	*Paenibacillus amylolyticus*	Accession: CP118896	[1]
Sp953	*Stenotrophomonas maltophilia*	Accession: CP118899	[1]
Sp1645	*Bacillus subtilis* 3610	Genotype: comI Q12I, DK1042	[2]
Sp1646	*Bacillus subtilis* DS1122	3610-derivate srfAC::Tn10spec	[3]

Searching for surfactant-related genes in *P. amylolyticus*

To search for homologues to the *srf*-operon *sfp* gene (origin: *B. subtilis* str. 168) (accession: X70356, X70357, X70358, X70359, ACG68433) in *P. amylolyticus* DR949 (accession: CP118896), we used blastp, with the *P. amylolyticus* genome as the query and the surfactin synthetase genes as the database. An e-value cutoff of 1e-5 and a conversative threshold of 30% similarity to assume homology between genes [4] was used.

Assessment of surfactant production

10 µL of overnight cultures of *P. amylolyticus* and *S. maltophilia* were plated onto Parafilm M. After two min, the shapes of the drops were imaged. A positive result was indicated by a spread out, or collapsed, drop (with *B. subtilis* as the positive control), whereas a negative result was indicated by a retained spherical shape (with. *B. subtilis* ΔsrfAC and TSB as the negative controls). To measure the external contact angle of the drops, ImageJ with the Contact Angle plugin was used. Two points were selected at the baseline of the drop; five points were selected to outline the shape of the drop. Using the manual points procedure, the theta e (for ellipses) value was used to obtain the contact angle of each drop.

Acquisition of transmission electron microscopy images

4 mL cultures of *P. amylolyticus* and *S. maltophilia* were grown overnight in 15 mL Falcon tubes at low shaking velocity. Immediately before sample preparation, tubes were gently inverted five times for mixing without damaging flagella. 4 µL were allowed to adsorb for 1 min on freshly glow discharged carbon grids (Ted Pella, Prod. No. 01840-F, Support films, Carbon, 200 mesh, Cu) and subsequently negatively stained with uranyl acetate 2% for 1 min. Samples were washed with ddH_2O to remove excess matrix and improve flagella visibility. The samples were examined with a Philips CM 100 TEM (Philips, Eindhoven, Netherlands), operated at an accelerating voltage of 80 kV. Digital images were recorded with an OSIS Veleta digital slow scan 2k x 2k CCD camera and the ITEM software package.

Swarming experiments

For the swarming experiments, overnight bacterial cultures were diluted and grown for 3 h until exponential phase. Then, the optical density was adjusted (OD_{600} = 0.05). When preparing dual-species samples, 100 µL of each respective OD-adjusted culture was added into a new Eppendorf tube. Swarming plates were prepared as 1.5% (W/V) TSB (Millipore; CAS 299092-500G) supplemented with 0.9% (W/V) Noble agar (Roth). After autoclaving, media was tempered to 60°C in a water bath for 1 h. Then, in a laminar bench, 25 mL of media was distributed into petri dishes (Ø=85 mm). After 10 min of drying with half the lid on, 3 µL of the OD_{600} adjusted cell cultures (mono- or dual-species samples) were inoculated onto the center of the plate. Swarming temporal dynamics were recorded using the Reshape Imaging System (RIS) robot (Reshape Biotech), acquiring images every 30 min at 24°C. Four biological replicates were done for each swarming condition.

To determine the optimal swarming agar percentage, *P. amylolyticus* was inoculated onto swarming plates with 1.5% (W/V) TSB supplemented with a range of Noble agar percentages (0.9, 1.1, 1.3) % (W/V). These plates were imaged every 30 min for 11 h. Image analysis was done with ImageJ to generate a heatmap of area (in mm^2) over time.

To identify which strains were present in the dual-species swarm after 72 h, the biomass at different swarm locations was restreaked onto selective antibiotic plates. The presence or absence of *P. amylolyticus* and *S. maltophilia* was tested by using antibiotic selection (streptomycin 5000 µg/mL for *P. amylolyticus* selection and erythromycin 15 µg/mL for *S. maltophilia* selection). Center refers to the swarm center, whereas side thick or side thin refer to the macroscopically thin or thick side of the swarm, respectively. Three biological replicates with two technical replicates were done to assess the species presence at different swarm locations.

Antibiotic susceptibility testing

Susceptibility to various antibiotics was assessed on a solid surface as clearing zones surrounding Oxoid discs as previously described [5]. For testing the antibiotic resistance profile of *P. amylolyticus* and *S. maltophilia*, 100 µL of overnight culture was mixed with 4 mL of 1.5% (W/V) TSB supplemented with 0.4% (W/V) agarose (VWR) and poured on top of a TSA plate (3% TSB, 1.5% agar, W/V). Once dry,

antibiotic discs of streptomycin (300 μg), tetracycline (30 μg), gentamycin (10 μg), chloramphenicol (30 μg), kanamycin (30 μg) or erythromycin (15 μg) were placed on top. Following 24 h of incubation at 24°C, clearing zones were measured. Three biological replicates were done for each antibiotic.

Assessing survival past antibiotic boundaries during swarming

Assessment of survival past chosen Oxoid antibiotic discs during swarming was conducted to assess the ability to cross barriers and exploit new areas. Swarming plates were poured as described above, but after six min, five disks of streptomycin (300 μg), erythromycin (15 μg), kanamycin (30 μg), tetracycline (30 μg), gentamycin (10 μg), or chloramphenicol (30 μg) were placed in a vertical line at the center of the plate. The bacteria were inoculated 2.5 cm from the line of antibiotics. The ability to swarm through these antibiotic discs was tested on *P. amylolyticus* as a monospecies swarm and on *P. amylolyticus* + *S. maltophilia*. The swarm surface area as a percentage of the entire plate was calculated using ImageJ after 16 h. Four biological replicates were done for the surface area calculations. To identify which member was able to swarm past the line of antibiotic, we sampled the biomass which crossed the antibiotic barrier with an inoculation loop and streaked on selective antibiotic plates. Streptomycin 5000 μg/mL was used for *P. amylolyticus* selection and erythromycin 15 μg/mL was used for *S. maltophilia*. Two biological replicates, with two technical replicates, were done for each species detection assay per antibiotic barrier.

Colony forming units (CFU) measurement of a swarm

Cell numbers were quantified for 12-, 24-, and 72-h old swarms. The entire swarm (or colony) biomass was collected with a cell scraper and inoculated into 1 mL of PBS. This was done for both mono- and two-mix swarms. The drop plate method was used for quantifying CFU/mL, where 10 μL of each sample was plated on antibiotic selective plates (streptomycin 5000 μg/mL for *P. amylolyticus* selection and erythromycin 15 μg/mL for *S. maltophilia* selection). For 12-h and 24-h old swarms, there were three biological replicates, each with three technical replicates. For 72-h swarms, six biological replicates, each with three technical replicates, were done to validate results.

Acquisition of confocal laser scanning microscopy (CLSM) images

72-h old mono and dual-species swarming plates were stained in situ using the *Bac*Light RedoxSensor Green vitality kit (Thermo Fisher) according to manufacturer's instructions. After 10 min of incubation in the dark, a scalpel was used to cut a 1 cm x 1 cm agar square, which was then placed upside down on a #1.5 cover glass (Thorlabs). Center refers to an agar square cut from the swarm center, whereas side thick or side thin refer to a square cut from the macroscopically thin or thick side of the swarm 25 mm from the swarm center. The stained agar piece was imaged with an inverted LSM800 confocal laser scanning microscope (Carl Zeiss) equipped with a 63x/1.40 Plan-Apochromat oil objective. Excitation of cells expressing green reductase activity was performed using a 488 nm laser, whereas a 561 nm laser was used for propidium iodide-stained cells. These images were used to calculate biofilm height

(defined as biovolume in the Z-direction). Height was calculated by multiplying the number of z stacks with the size of respective z stack in the ZEN blue software (Carl Zeiss Inc.). Two biological replicates with at least two technical replicates were done.

pH assessments of planktonic cultures and swarms

We measured the pH over time for planktonic cultures of *P. amylolyticus*, *S. maltophilia*, as well as *P. amylolyticus* + *S. maltophilia*. 50X dilutions of overnight cultures were inoculated into 100 mL of TSB in Erlenmeyer flasks (300 mL size) and placed at 24 °C. pH was recorded at time points 0.0, 2.5, 5.0, 25.5, and 29.0 h. Two biological replicates were done. To visualize the pH overtime in situ in the swarm plates, 0.018 g/L of phenol red indicator was added to the swarming media.

Buffering swarming plates to pH 5.0 and pH 6.7

To determine whether pH modulation was the key determinant of *P. amylolyticus* survival in dual-species swarms, 100 mM MES hydrate (Sigma M2933) was used as a pH buffer; the pH of the swarming plates was adjusted to pH = 5.0 and pH = 6.7, using 10M NaOH (Sigma) and 6M HCl (Supelco) solutions, respectively. The swarming assay and subsequent quantification of survival was done as described above. Three biological replicates with three technical replicates were done.

Reculturing three-day old *P. amylolyticus* swarms with pH assay

TSB solutions (3% W/V) at different pHs (3.5, 4.5, 5.5, 6.5, 7.5) were made. Streaks from *P. amylolyticus* monospecies swarms and dual-species (*P. amylolyticus* + *S. maltophilia*) swarms were tested for their ability to grow. Three-day old swarms were scraped into 1 mL of PBS and vortexed thoroughly. We added 3 µL of this culture to a total of 150 µL of TSB in each well. Streptomycin 5000 µg/mL was added to the media to select for *P. amylolyticus* in both conditions (mono- and dual-species swarms). The cultures were then grown in a microtiter plate for 48 h with continuous, double orbital shaking at 237 cpm. OD_{600} was measured over time. Two biological replicates with three technical replicates each were done.

Supplementary figures

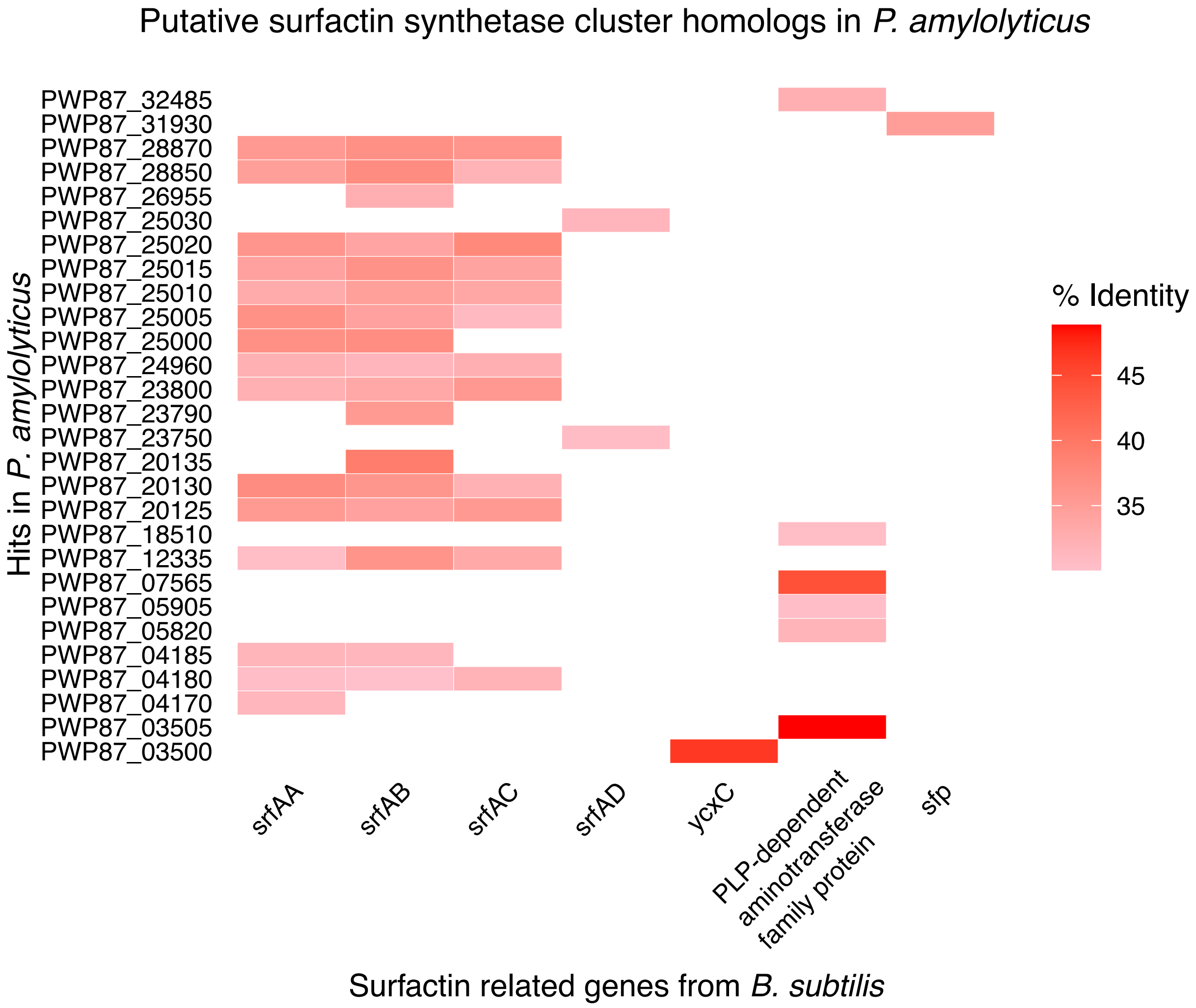

Supplementary Figure 1. Genomic identification in *P. amylolyticus* of genes involved in surfactin production. The *srfA* operon-*sfp* gene cluster from *Bacillus subtilis* was used as the query for the blastp search against the *Paenibacillus amylolyticus* DR949 genome. Matching hits are shown in red, with a darker color indicating greater similarity. The e-value cutoff was 1e-5. Using the conservative cutoff of 30% similarity to infer homology between genes [4], we can see there are various hits in the *P. amylolyticus* genome which could potentially encode for the genes involved in surfactin synthetase.

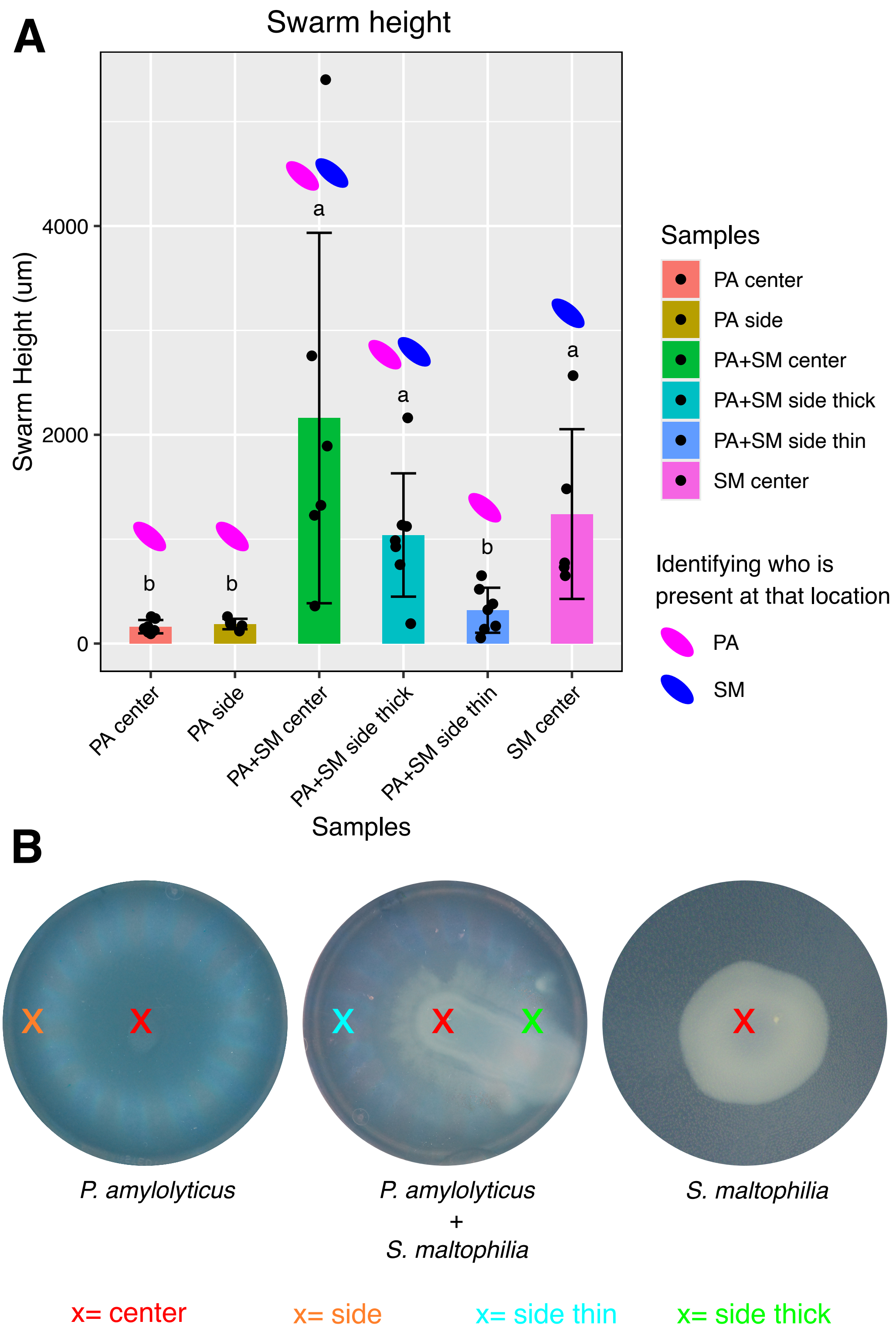

Supplementary Figure 2. Comparative heights of mono- and dual-species swarms. A) Heights of *Paenibacillus amylolyticus* (PA), *Stenotrophomonas maltophilia* (SM), and *P. amylolyticus* + *S. maltophilia* (PA + SM) swarms were measured after 72 h using *Bac*Light RedoxSensor vitality and propidium iodide to stain all cells. Height is defined as biovolume in the Z-direction. Center refers to the swarm center, whereas side

thick or side thin refer to the macroscopically thin or thick side of the swarm 25 mm from the swarm center. Biofilm height was statistically different of PA center, PA side, and PA+SM side thin (indicated by b) compared to PA+SM center, PA+SM side thick, and SM center (indicated by a) (one way ANOVA with Tukey's multiple comparisons). To assess which of the two bacterial members were present in different areas of the swarm, we streaked the biomass at the respective locations and tested for the presence of each species using selective plating (streptomycin 5000 µg/mL for *P. amylolyticus* and erythromycin 15 µg/mL for *S. maltophilia*). *P. amylolyticus* is indicated as a pink oval and *S. maltophilia* as a blue oval. **B)** Representative images of the respective swarms and sampling locations are shown. A red X indicates the swarm center, an orange X indicates a side, a blue X indicates a thin side, and a green X indicates a thick side.

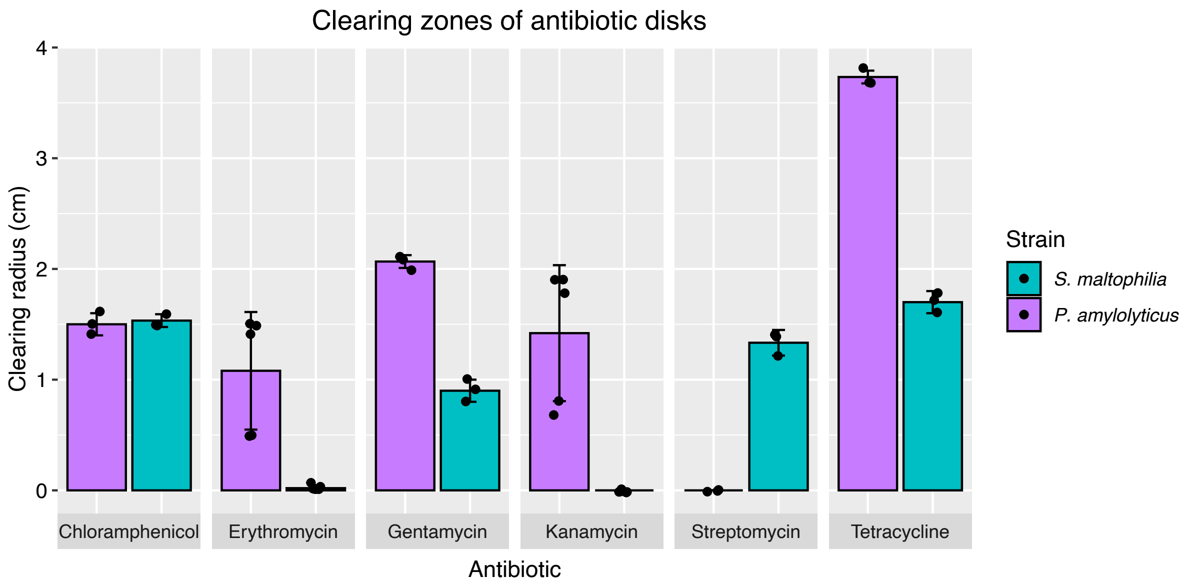

Supplementary Figure 3. Antibiotic resistance measured as clearing zones of a bacterial lawn. Antibiotic clearing zones around streptomycin (300 µg), tetracycline (30 µg), gentamycin (10 µg), chloramphenicol (30 µg), erythromycin (15 µg) and kanamycin (30 µg) antibiotic Oxoid discs. Clearance diameters were measured and matched previous results [5]. Clearing zones (mm) were recorded for *Paenibacillus amylolyticus* (PA) and *Stenotrophomonas maltophilia* (SM). Three biological replicates were done for each antibiotic.

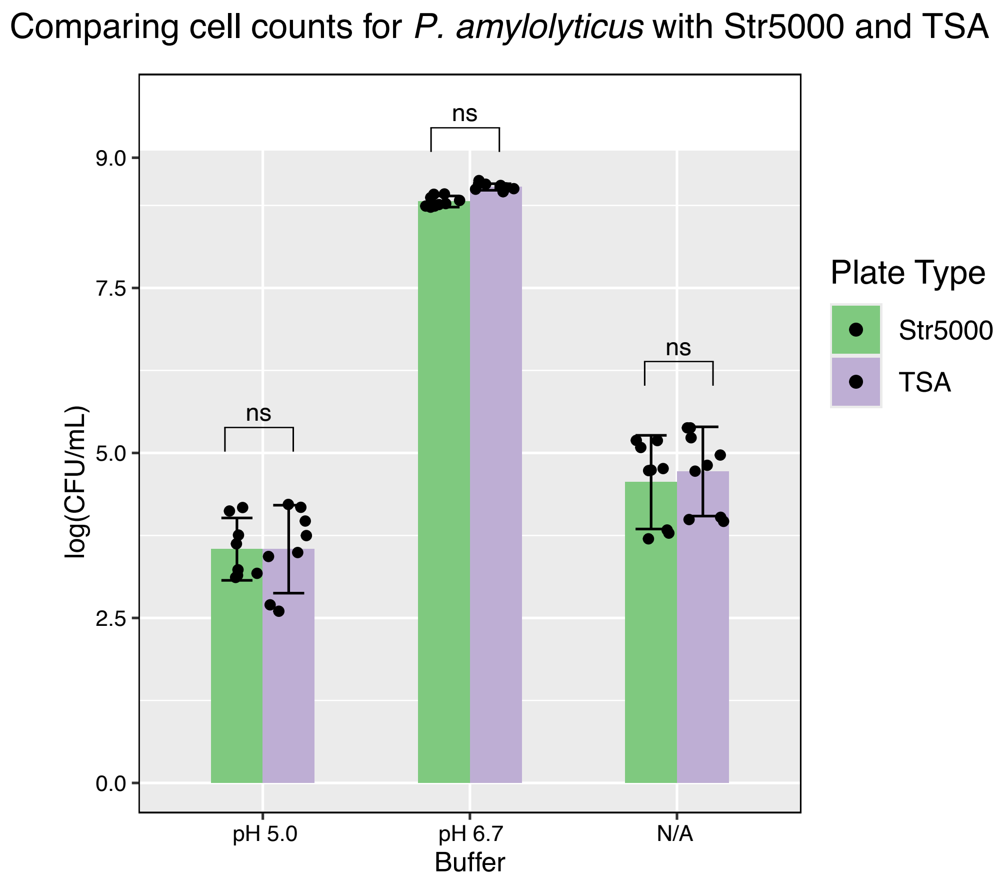

Supplementary Figure 4. Attesting antibiotic selection for *P. amylolyticus*. To ensure that streptomycin 5000 µg/mL was not impacting the number of *P. amylolyticus* cells able to regrow, we compared counts for three-day old *P. amylolyticus* swarms on TSA and on TSA supplemented with streptomycin 5000 µg/mL. We also tested the effect of first buffering the swarming plates (pH 5.0 and 6.7) with a MES buffer and then plating on TSA or TSA supplemented with streptomycin 5000 µg/mL. There was no difference between counts when comparing the two respective selection regimes (two-sample t-test).

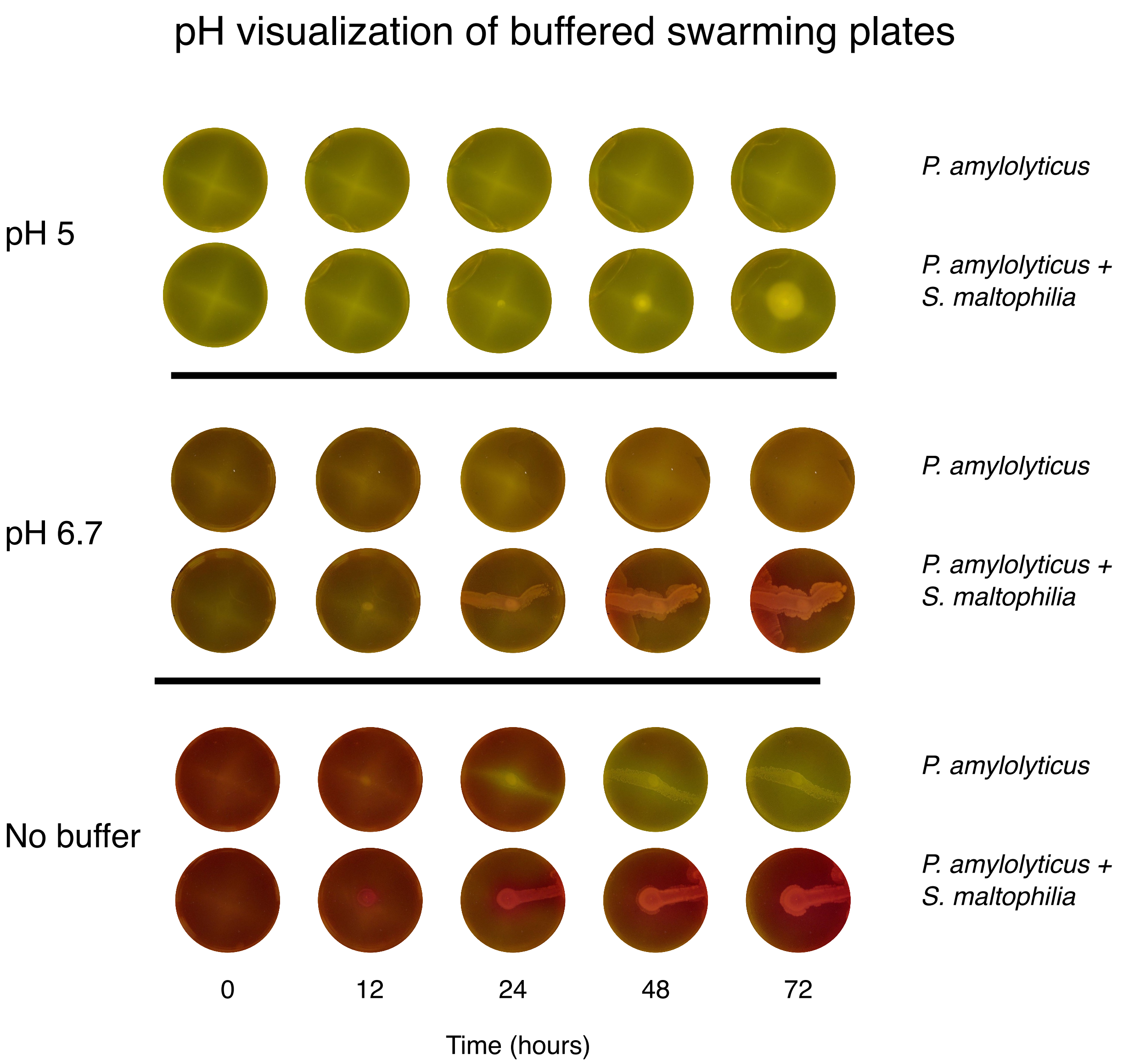

Supplementary Figure 5. Testing the efficacy of the pH buffer with phenol red pH indicator. Swarming plates were buffered to pH 5.0 and pH 6.7. To test the efficacy of the buffer, phenol red pH indicator was added to the plates. Images for mono- and dual-species swarms are shown over time, in swarming plates buffered to pH 5.0, pH 6.7, or with no buffer.

Bibliography

[1] Ren D, Madsen JS, de la Cruz-Perera CI, et al. High-throughput screening of multispecies biofilm formation and quantitative PCR-based assessment of individual species proportions, useful for exploring interspecific bacterial interactions. Microb Ecol 2014;68:146–54. https://doi.org/10.1007/s00248-013-0315-z.

[2] Konkol MA, Blair KM, Kearns DB. Plasmid-encoded ComI inhibits competence in the ancestral 3610 strain of *Bacillus subtilis*. J Bacteriol 2013;195:4085–93. https://doi.org/10.1128/JB.00696-13.

[3] Chen R, Guttenplan SB, Blair KM, et al. Role of the sigmaD-dependent autolysins in *Bacillus subtilis* population heterogeneity. J Bacteriol 2009;191:5775–84. https://doi.org/10.1128/JB.00521-09.

[4] Pearson WR. An Introduction to sequence similarity (“homology”) searching. Curr Protoc Bioinform 2013; Jun:Chapter 3:3.1.1-3.1.8. https://doi.org/10.1002/0471250953.bi0301s42. https://doi.org/10.1002/0471250953.bi0301s42.

[5] Ronin D, Hansen MF, Flaig ML, et al. Unfolding the collective functional potential of a synergistic multispecies community through genotypic and phenotypic analyses. Biofilm 2025;10:100290. https://doi.org/10.1016/j.bioflm.2025.100290.
